# The mediating role of physical activity attitude in the relationship between physical literacy and a sustainable healthy lifestyle in adolescents

**DOI:** 10.3389/fpsyg.2025.1661080

**Published:** 2025-10-29

**Authors:** İsmail İlbak, Mehmet Akarsu, Ladislav Cepicka, Petr Valach

**Affiliations:** ^1^Institute of Health Sciences, İnönü University, Malatya, Türkiye; ^2^Department of Physical Education and Sports, İnönü University, Malatya, Türkiye; ^3^Department of Physical Education and Sport, University of West Bohemia, Pilsen, Czechia

**Keywords:** physical literacy, sustainable healthy lifestyle, physical activity attitude, adolescents, perceived physical literacy instrument for adolescents, lifestyle of health and sustainability scale

## Abstract

**Background and aim:**

The sustainability of individuals’ health is not solely dependent on medical interventions but also on the adoption of healthy lifestyle behaviors. The aim of this study was to examine the effect of physical literacy on sustainable healthy lifestyle behaviors in adolescents and to investigate the mediating role of physical activity attitude in this relationship.

**Methods:**

Physical literacy was defined as the independent variable, sustainable healthy lifestyle as the dependent variable, and physical activity attitude as the mediator. Data were collected from a total of 750 voluntary participants (mean age = 15.71 ± 0.99) using the Perceived Physical Literacy Instrument for Adolescents (PPLI), the Lifestyle of Health and Sustainability Scale (LOHAS), and the Youth Physical Activity Attitude Scale (YPAAS). The total score and subscale interpretations of these instruments were considered in the analyses. Ethical approval was obtained, and informed consent was secured from guardians, as well as assent from the adolescents. Data were analyzed using correlation, regression, and structural equation modeling (SEM), with a significance threshold set at *p* < 0.05.

**Results:**

The findings revealed a significant and positive relationship between physical literacy and a sustainable healthy lifestyle (*r* = 0.484, *p* < 0.001). Physical activity attitude was also significantly correlated with both physical literacy (*r* = 0.447, *p* < 0.001) and sustainable healthy lifestyle (*r* = 0.400, *p* < 0.001). SEM results indicated that physical literacy directly (*β* = 0.519) and indirectly (*β* = 0.139) through physical activity attitude significantly influenced sustainable healthy lifestyle behaviors. The model explained 28% (*R*^2^ = 0.28) of the variance in sustainable healthy lifestyle behaviors.

**Conclusion:**

These results highlight that physical activity attitude plays a partial mediating role in the relationship and demonstrate that physical literacy is not merely a cognitive or skill-based construct, but a comprehensive competency that shapes adolescents’ attitudes and behaviors. Accordingly, education and health policies aimed at promoting physical literacy should be supported by holistic strategies that also target attitude transformation. The findings contribute to the theoretical literature and highlight the importance of multifaceted intervention models in promoting healthy lifestyle behaviors among adolescents.

## 1 Introduction

Recent research indicates that maintaining and enhancing individuals’ quality of life is achievable not only through medical interventions but also by adopting healthy lifestyles (Das, 2025; Safeer and Lucik, 2023). A healthy lifestyle encompasses a range of multidimensional behaviors, including regular physical activity, balanced nutrition, sufficient sleep, stress management, and avoidance of harmful habits (Grover et al., 2023). Adolescence, in particular, represents a critical developmental phase during which lifelong health-related habits are established. The adoption of healthy lifestyle behaviors during this period plays a decisive role in determining an individual’s future health status and quality of life (Chaku and Davis-Kean, 2024; Rowland, 2007). However, the sustainability of a healthy lifestyle is not only linked to behavioral choices but also to the cognitive, affective, and behavioral components that drive these choices (Akhtar, 2023; Kang et al., 2023; Sultan and Nag, 2020). In this context, the concept of physical literacy has increasingly gained attention at the intersection of health and education, referring to the knowledge, skills, attitudes, and motivation necessary for individuals to lead active lives.

According to the International Physical Literacy Association (2025) (IPLA), physical literacy is defined as “the motivation, confidence, physical competence, knowledge, and understanding to value and take responsibility for engaging in physical activities for life.” This holistic perspective recognizes four widely accepted pillars of physical literacy: (i) affective (motivation and confidence), (ii) physical (competence and motor skills), (iii) cognitive (knowledge and understanding), and (iv) behavioral/participation (drive and opportunities for engagement across the lifespan). Situating physical literacy within its monist philosophical roots with phenomenological and existential influences further underscores that movement is conceived as an embodied experience. Thus, physical literacy should not be viewed merely as the sum of “skills+knowledge” but as an integrated competence that sustains participation and engagement throughout life.

Building on this holistic definition, physical literacy provides the foundation for individuals’ participation in physical activities by integrating motor skills, physical competence, motivation, confidence, and knowledge, thereby supporting lifelong engagement in physical activity (Nurjanah et al., 2025). This ontological framing directly supports the rationale for the present study’s hypotheses and clarifies why attitudes particularly toward physical activity are expected to mediate the pathway between physical literacy and sustainable healthy lifestyle behaviors. Evidence supporting the notion that physical literacy promotes greater participation in physical activities, positively influences healthy lifestyle behaviors, and supports psychosocial well-being is growing in the literature (Carl et al., 2022; Fang and Zhang, 2024; Melby et al., 2022; Urbano-Mairena et al., 2023). Nevertheless, to better understand the relationship between physical literacy and a sustainable healthy lifestyle, it is necessary to analyze potential mediating variables. Investigating the mediating role of physical activity attitude in this relationship is crucial for both deepening the theoretical framework and developing practical strategies.

An individual’s attitude toward physical activity is considered a key variable in sustaining this behavior (Kudubes et al., 2022). Physical activity attitude can be defined as an individual’s cognitive, affective, and behavioral predisposition toward engaging in physical activity, reflecting beliefs, emotions, and tendencies that influence participation (Ajzen, 1991; Graham et al., 2011). Positive attitudes toward physical activity increase the likelihood of maintaining regular participation, whereas negative attitudes adversely affect engagement and continuity (Kudubes et al., 2022; Szakály et al., 2017). Particularly for adolescents, factors such as social environment, self-confidence, achievement perceptions, and body image significantly influence their attitudes toward physical activity (Graham et al., 2011; Mabry et al., 2003; Oja and Piksööt, 2022). Therefore, it is essential to investigate whether physical literacy fosters positive attitudes toward physical activity and, in turn, contributes to the sustainability of a healthy lifestyle.

The present study aims to examine the effect of physical literacy on the sustainable healthy lifestyle of adolescents and to explore the mediating role of physical activity attitude in this relationship. Accordingly, it is hypothesized that physical literacy has a direct and positive effect on sustainable healthy lifestyle, that physical literacy positively influences physical activity attitude, and that physical activity attitude has a direct and positive effect on sustainable healthy lifestyle. It is also hypothesized that physical activity attitude partially mediates the relationship between physical literacy and sustainable healthy lifestyle. It is expected that the findings will contribute to the theoretical knowledge base while also providing guidance for education policies, health programs, and intervention strategies targeting youth.

## 2 Methodology

### 2.1 Study group

In this study, the required minimum sample size was determined using the G*Power software (version 3.1.9.7; University of Düsseldorf, Düsseldorf, Germany). The power analysis was based on an effect size of 0.03, a significance level (*α*) of 0.05, a statistical power (1-*β*) of 0.95, and a predictor count of 3. Based on these parameters, the minimum number of participants required was calculated to be 577. To exceed the recommended threshold and enhance statistical robustness, a total of 750 participants were voluntarily included in the study. The sample was selected through purposive sampling. Participants consisted of high school students enrolled in formal education in various schools across Türkiye, who were actively attending school during the data collection period and voluntarily agreed to participate in the study. To be included in the sample, individuals had to be between 14 and 17 years of age, be registered as full-time students in formal education institutions, be cognitively and physically capable of completing the data collection instruments, and provide informed assent. Given that participants were under the age of 18, written informed consent was also obtained from their parents or legal guardians. Individuals with severe physical or cognitive impairments, incomplete or inaccurate responses to measurement tools, or who failed to complete the data collection process were excluded from the study. The research process was conducted in full compliance with the ethical principles outlined in the Declaration of Helsinki and relevant ethical standards. Throughout the study, all personal data were handled with strict confidentiality. Detailed demographic characteristics of the participants are presented in Table 1.

**Table 1:** Demographic characteristics of the participants.

Variable		*x̄*	SD
Age (years)		15.71	0.99
Height (cm)		170.24	9.12
Weight (kg)		60.94	12.38
		*n*	%
Gender	Male	339	45.20
	Female	411	54.80

*X̄*, mean; SD, standard deviation.

Table 1 presents the demographic characteristics of the study sample, which consisted of 750 adolescents (54.8% female, *n* = 411; 45.2% male, *n* = 339). The participants’ mean age was 15.71 years (SD = 0.99), mean height was 170.24 cm (SD = 9.12), and mean weight was 60.94 kg (SD = 12.38).

### 2.2 Research model

**Figure 1:**
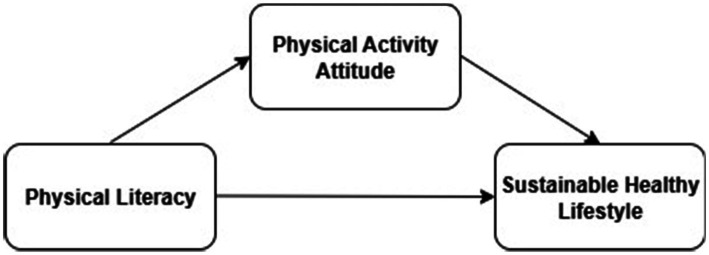
Structural model of the relationships among physical literacy, physical activity attitude, and sustainable healthy lifestyle.

This study was conducted within the framework of a relational survey design. In the theoretical model, physical literacy was conceptualized as the independent variable, sustainable healthy lifestyle as the dependent variable, and attitude toward physical activity as the mediating variable. The model aimed to explain the potential causal relationships among these variables and to empirically test them using Structural Equation Modeling (SEM). The analysis was based on data obtained from 750 high school students who met the inclusion criteria. Ethical approval for the study was obtained from the Social and Humanities Research and Publication Ethics Committee of İnönü University (Approval No: 17; Date: April 28, 2025). The research was conducted in full compliance with ethical principles, and all responsibilities concerning participant rights and research integrity were rigorously upheld. The model is presented in Figure 1, and the study sought to evaluate the hypothesized direct and indirect effects among the specified constructs.

### 2.3 Data collection tools

The data collection tools used in this study were carefully structured to minimize the cognitive load on participants and to ensure that the response process progressed naturally, meaningfully, and in a motivating flow. The sequence of scale administration followed a gradual structure, moving from general to more specific and cognitively demanding content. In the initial stage, a Demographic Information Form was administered to collect basic demographic data such as age, gender, height, and weight. Following this, participants’ general attitudes toward physical activity were assessed using the Youth Physical Activity Attitude Scale. In the second stage, deeper cognitive constructs related to subjective evaluations and individual awareness of physical activity were targeted by administering the Perceived Physical Literacy Instrument for Adolescents. Finally, the Lifestyle of Health and Sustainability Scale was used to comprehensively assess not only health behaviors but also environmental awareness, sensitivity to sustainability, and lifestyle preferences. This structured flow aimed to maintain participants’ interest and motivation throughout the process, ensuring a natural continuity of responses and enhancing the reliability of the data collection procedure.

#### 2.3.1 Lifestyle of Health and Sustainability Scale

In this study, the Lifestyle of Health and Sustainability Scale, originally developed by Choi and Feinberg (2021) and adapted into Turkish by Gökkaya (2024), was employed to assess individuals’ sustainable healthy lifestyle behaviors. The original version of the scale consists of 28 items; however, the Turkish adaptation includes 24 items. The scale uses a 5-point Likert-type response format ranging from 1 (“Strongly Disagree”) to 5 (“Strongly Agree”). The Lifestyle of Health and Sustainability Scale consists of six sub-dimensions: physical fitness, mental health, emotional health, spiritual health, environmentalism, and social awareness. The minimum score obtainable from the scale is 24, while the maximum score is 120. Higher scores reflect a healthier and more sustainable lifestyle, whereas lower scores indicate less healthy and sustainable lifestyle behaviors. In the Turkish adaptation study conducted by Gökkaya, the internal consistency of the scale was reported with a Cronbach’s alpha coefficient of 0.895. Confirmatory factor analysis (CFA) conducted in the same adaptation study indicated an acceptable model fit, with the following indices: *χ*^2^/df = 1.895, CFI = 0.950, TLI = 0.941, GFI = 0.909, IFI = 0.950, and RMSEA = 0.050. In the current study, the Cronbach’s alpha internal consistency coefficient was calculated as 0.717. CFA conducted using the present sample also confirmed the structural validity of the Turkish version of the scale, yielding satisfactory fit indices: *χ*^2^/df = 2.474, CFI = 0.921, TLI = 0.904, GFI = 0.985, IFI = 0.922, and RMSEA = 0.047. These results indicate that the scale is a valid and reliable tool for assessing sustainable healthy lifestyle behaviors in Turkish adolescents.

#### 2.3.2 Perceived Physical Literacy Instrument for Adolescents

To assess adolescents’ levels of physical literacy, the Perceived Physical Literacy Instrument for Adolescents, developed by Sum et al. (2018) and adapted into Turkish by Yılmaz and Kabak (2021), was utilized in this study. The scale consists of 9 items and employs a 5-point Likert-type format (1 = Strongly Disagree, 5 = Strongly Agree). The Perceived Physical Literacy Instrument for Adolescents consists of three sub-dimensions: Knowledge and Understanding, Self-expression and Communication with Others, and Sense of Self and Self-confidence. The minimum score obtainable from the scale is 9, while the maximum score is 45. Higher scores indicate higher levels of perceived physical literacy among adolescents. In the Turkish adaptation study conducted by Yılmaz and Kabak, the scale demonstrated high internal consistency, with a Cronbach’s alpha coefficient of 0.900. Confirmatory factor analysis (CFA) results from the same adaptation study indicated an acceptable model fit, with the following fit indices reported: *χ*^2^ = 146.326, df = 69, CFI = 0.97, TLI = 0.97, RMSEA = 0.061, and SRMR = 0.082. These results provided strong support for the structural validity of the Turkish version of the scale. In the current study, the scale also showed satisfactory internal consistency (Cronbach’s alpha = 0.811). CFA conducted with the present sample yielded the following fit indices: *χ*^2^/df = 4.28, CFI = 0.963, TLI = 0.942, RMSEA = 0.066, and SRMR = 0.034. These findings confirm that the Turkish version of the scale retains a strong structural validity when applied to the current adolescent sample.

#### 2.3.3 Youth Physical Activity Attitude Scale (YPAAS)

The Youth Physical Activity Attitude Scale (YPAAS), developed by Simonton et al. (2021) and adapted into Turkish by Uyhan et al. (2023), was used in this study to assess children’s and adolescents’ attitudes toward physical activity. The scale consists of 12 items and is rated on a 5-point Likert-type scale (1 = Definitely No, 5 = Definitely Yes). The YPAAS includes two sub-dimensions: Positive Attitudes (seven items; score range 7–35) and Negative Attitudes (5 items; score range 5–25). Higher scores on the positive subscale indicate more favorable attitudes toward physical activity, whereas higher scores on the negative subscale reflect more unfavorable attitudes. In the interpretation of the overall scale, the subscales are typically examined separately, since summing the two dimensions may obscure differences (e.g., high positive and high negative scores could yield similar totals). In this study, we calculated the mean scores for each sub-dimension separately to provide a more accurate interpretation of adolescents’ attitudes toward physical activity. This approach allowed us to distinguish between positive and negative attitudes rather than relying solely on a combined score. The original scale demonstrated high internal consistency, with a Cronbach’s alpha coefficient reported to be above 0.800. In the present study, the internal consistency of the Turkish version was also found to be high, with a Cronbach’s alpha of 0.852. Confirmatory factor analysis (CFA) results reported in the Turkish adaptation study indicated an acceptable model fit, with the following indices: *χ*^2^/df = 1.55, CFI = 0.97, GFI = 0.97, NFI = 0.93, IFI = 0.97, and RMSEA = 0.040. In the current study, CFA was performed again to validate the scale’s structure within the sample, and the following fit indices were obtained: *χ*^2^/df = 3.69, CFI = 0.94, GFI = 0.99, NFI = 0.92, IFI = 0.94, and RMSEA = 0.060. These results provide strong evidence supporting the structural validity of the Turkish version of the scale when applied to the current adolescent population.

### 2.4 Data collection procedure

Participants were informed in advance about the purpose, scope, and confidentiality principles of the research, and the data collection process was conducted entirely on a voluntary basis. Data were collected between May 12 and May 16, 2025, in classroom settings at high schools, under the direct supervision of trained researchers experienced in adolescent research and field implementation. All data collection took place during regular class hours but outside of examination periods, in order to minimize distractions and psychological stress. Prior to participation, an informed consent form was presented to all students, clearly stating that participation was voluntary, that responses would be used exclusively for scientific purposes, and that all personal information would be kept strictly confidential. Since the participants were under the age of 18, an information letter explaining the study’s aims, scope, ethical principles, and confidentiality procedures, together with a written informed consent form, was sent to parents/legal guardians via the school administration. Only students whose parents/legal guardians signed and returned the consent forms were allowed to participate in the study. In addition, adolescents themselves provided informed assent before completing the questionnaires. Participants were explicitly reminded that there were no right or wrong answers and were encouraged to respond honestly to reduce social desirability bias. The data collection was conducted using a structured questionnaire form developed in line with the study’s objectives. Demographic information was collected first, followed by the administration of the measurement instruments in a predetermined sequence. To ensure data completeness and integrity, all scale items were required to be answered. The average duration of participation was approximately 15 min per respondent. Special attention was paid to maintaining participant motivation, particularly due to the relatively lengthy structure of the Lifestyle of Health and Sustainability Scale (24 items) in combination with the other instruments. The data collection environment was kept quiet, organized, and free from distractions. Clear verbal and written instructions were provided to ensure participants fully understood the process, reduce fatigue, and enhance response accuracy. All responses were anonymized and were not linked to any identifying information. The collected datasets were securely stored in digital environments and used solely for the purposes of this study.

## 3 Data analysis

The analysis of the data obtained from the study was conducted using JASP statistical software (version 0.18.3.0; University of Amsterdam, Amsterdam, the Netherlands). The normality of the data distribution was assessed by examining skewness and kurtosis values, ensuring they fell within the ±2 range (Kim, 2013; Mishra et al., 2019; Tabachnick and Fidell, 2019). The analyses confirmed that the data met the assumption of normal distribution. In the calculation of the total score for the Youth Physical Activity Attitude Scale (YPAAS), the total score of the Negative Attitudes sub-dimension was reverse-coded, and then combined with the Positive Attitudes sub-dimension to obtain the overall Physical Activity Attitude (PAA) score used in the analyses. Accordingly, Pearson correlation analysis was conducted to determine the relationships among the variables. Before structural equation modeling (SEM), several assumptions were evaluated. Linearity was visually checked through scatterplots. Multicollinearity was assessed via tolerance and variance inflation factor (VIF) values, all of which remained within acceptable limits (VIF < 5, Tolerance > 0.20) (Hair et al., 2014).

In the research model, physical literacy was positioned as the independent variable, sustainable healthy lifestyle as the dependent variable, and attitude toward physical activity as the mediating variable. To test the statistical significance of the mediation effect, a bootstrapping method with 5,000 resamples was employed. The resulting 95% confidence intervals were examined to evaluate the significance of indirect effects; the absence of zero within these intervals indicated statistically significant mediation effects (Preacher & Hayes, 2008). In addition to bootstrap-ping, *z*-values were also reported (Table 2) to further support the robustness of the mediation analysis. For all statistical analyses, a significance threshold of *p* < 0.05 was adopted.

**Table 2:** Findings on direct, indirect and mediating effects.

Direct effects			%95 Confidence interval							
					*β*	Std. error	*z*-value	*p*	Lower	Upper
PAA	→	SHL			0.229	0.035	6.588	< 0.001	0.161	0.297
PL	→	SHL			0.519	0.047	11.001	< 0.001	0.427	0.611
PL	→	PAA			0.608	0.044	13.697	< 0.001	0.521	0.695

Indirect effects			%95 Confidence interval							
					*β*	Std. error	*z*-value	*p*	Lower	Upper
PL	→	PAA	→	SHL	0.139	0.023	5.937	< 0.001	0.093	0.185

Total effects			%95 Confidence interval							
					*β*	Std. error	*z*-value	*p*	Lower	Upper
PL	→	SHL			0.658	0.043	15.163	< 0.001	0.573	0.743

The variance explained	*R*^2^
SHL	0.276
PAA	0.200

PL, physical literacy; SHL, sustainable healthy lifestyle; PAA, physical activity attitude.

## 4 Results

The findings obtained within the scope of the study are presented below through tables and figures. In this context, the relationships between physical literacy, sustainable healthy lifestyle, and physical activity attitude are shown in Table 3.

**Table 3:** Means, standard deviations, skewness, kurtosis, and correlations among study variables.

Variables	PL	SHL	PAA	*x̄*	SD	Skewness	Kurtosis
PL	–	0.484**	0.447**	3.72	0.74	−0.626	0.353
SHL	–	–	0.400**	3.20	0.57	−0.368	0.881
PAA	–	–	–	3.53	0.74	−0.483	0.366

PL, physical literacy; SHL, sustainable healthy lifestyle; PAA, physical activity attitude. **p < 0.001.

As shown in Table 3, the mean score of SHL was 3.20 (SD = 0.57), PL was 3.72 (SD = 0.74), and PAA was 3.53 (SD = 0.74). A significant positive correlation was found between PL and SHL (*r* = 0.484, *p* < 0.001). There was also a significant positive correlation between PL and PAA (*r* = 0.447, *p* < 0.001). Furthermore, SHL and PAA were significantly positively correlated (*r* = 0.400, *p* < 0.001).

As presented in Table 2, the mediation analysis results indicate that the direct effect of physical activity attitude (PAA) on sustainable healthy lifestyle (SHL) was statistically significant [*β* = 0.229, z = 6.588, 95% CI (0.161, 0.297), *p* < 0.001], representing a small-to-moderate effect size. The direct effect of physical literacy (PL) on SHL was also significant [*β* = 0.519, *z* = 11.001, 95% CI (0.427, 0.611), *p* < 0.001], corresponding to a large effect size. Likewise, the direct effect of PL on PAA was statistically significant [*β* = 0.608, z = 13.697, 95% CI (0.521, 0.695), *p* < 0.001], also indicating a large effect size.

In terms of indirect effects, the effect of PL on SHL through PAA was significant [*β* = 0.139, *z* = 5.937, 95% CI (0.093, 0.185), *p* < 0.001], reflecting a small-to-moderate effect size. Regarding total effects, the overall effect of PL on SHL was statistically significant [*β* = 0.658, *z* = 15.163, 95% CI (0.573, 0.743), *p* < 0.001], interpreted as a large effect size.

The variance explained (*R*^2^) by PL and PAA on SHL was 28%, while PL explained 20% of the variance in PAA.

**Figure 2:**
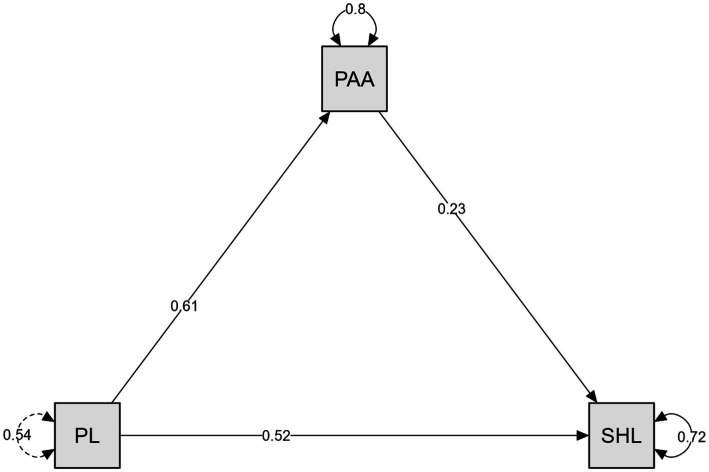
Mediation model of physical activity attitude between physical literacy and sustainable healthy lifestyle.

Figure 2 presents the structural equation model that investigates the mediating role of physical activity attitude (PAA) in the relationship between physical literacy (PL) and sustainable healthy lifestyle (SHL). As shown in the model, the direct effect of PL on PAA is positive (*β* = 0.61). Similarly, the direct effect of PL on SHL is positive (*β* = 0.52). Additionally, the direct effect of PAA on SHL is also positive (*β* = 0.23). The values placed inside the squares (PL = 0.54; PAA = 0.80; SHL = 0.72) do not represent *R*^2^ coefficients; rather, they indicate the internal consistency reliability of the constructs within the model. The actual R^2^ values are reported separately in Table 2.

## 5 Discussion

This study was conducted to examine the effect of physical literacy on sustainable healthy lifestyle among adolescents and to determine the mediating role of physical activity attitude in this relationship. The findings reveal that the structure of these relationships is both significant and meaningful.

A significant and positive relationship was found between physical literacy and sustainable healthy lifestyle. This finding is consistent with Whitehead’s (2010) definition of physical literacy as a construct that supports lifelong participation in physical activity. Similarly, Caldwell et al. (2020) also found a positive association between physical literacy and healthy lifestyle behaviors among young individuals. These findings align with the conceptual model proposed by Cairney et al. (2019), which positions physical literacy as a key determinant of health through its influence on both physical behavior and biological systems. These results confirm that physical literacy contributes not only to immediate physical engagement but also to long-term health-promoting behaviors. By emphasizing physical literacy as an integrated competence rather than a simple sum of knowledge and skills, the study underscores its philosophical grounding and its relevance to sustainable health behaviors. These results suggest that physical literacy is not merely a set of knowledge and skills but a comprehensive competence that directs behaviors. In particular, it encompasses cognitive, affective, and motivational dimensions, all of which are essential in shaping lifestyle choices in adolescence a period marked by habit formation and identity development (Steinberg, 2014; Sawyer et al., 2012).

The significant positive relationship between physical literacy and physical activity attitude is also noteworthy. As demonstrated in the study by Öztürk et al. (2023), increases in physical literacy levels positively influence both participation in physical activities and attitudes toward them. Furthermore, according to Ajzen’s (1991) Theory of Planned Behavior, attitude is considered a critical antecedent in guiding individual behavior, supporting the idea that physical literacy plays a fundamental role in transforming attitudes into behavior. This suggests that strengthening physical literacy can foster more favorable attitudes toward physical activity, which in turn may support healthier lifestyle choices an interpretation consistent with both theory and prior evidence.

One of the most important findings of the study is that physical activity attitude serves as a significant mediator between physical literacy and sustainable healthy lifestyle. This highlights that the impact of physical literacy on behavioral outcomes largely occurs through affective components. Woolley et al. (2024) reported that positive emotions are associated with physical literacy, with 73% of students experiencing such emotions during physical education classes. Similarly, Melby et al. (2022) and Blain et al. (2020) emphasized that physical literacy positively influences physical activity behaviors, with attitudes and emotional processes playing a key role. These findings underline the importance of positive physical activity attitudes in forming sustainable healthy living habits. In other words, without the development of a positive attitude, physical knowledge and skills alone may not translate into desired health behaviors. Thus, a positive attitude toward physical activity can be considered a critical bridge for adolescents in adopting a healthy lifestyle. This mediating mechanism underscores the importance of targeting emotional and motivational variables in health promotion programs, particularly for younger populations who are still developing self-regulation and goal-setting capacities (Steinberg, 2014; Gestsdottir and Lerner, 2008).

Moreover, the variance explained by the model is remarkable. The fact that physical literacy and physical activity attitude together explain 28% of the variance in sustainable healthy lifestyle indicates the importance of evaluating these two constructs together. While this percentage reflects a moderate level of explanatory power, it also implies that other factors such as family support (Tabrizi et al., 2024), peer influence (Yang, 2024), environmental access (Zsákai et al., 2023), and digital media use (Bustomi and Prihayati, 2024) may contribute significantly to adolescents’ health behaviors and should be examined in future studies. Additionally, exploring potential differences across subgroups such as gender using metric or scalar invariance testing in future research could provide further insights into the robustness of these relationships. This proportion suggests that educational programs should not be limited to knowledge transfer but should also include content aimed at developing positive attitudes. Furthermore, implementing experiential and emotionally engaging physical education curricula may amplify the impact of such programs, leading to more enduring behavioral outcomes.

In this regard, the current study not only fills a theoretical gap but also provides important insights for practical applications. Health and education programs targeting youth may increase their effectiveness by incorporating both the development of physical literacy and the structures that shape individual attitudes. Specifically, a dual focus on skill acquisition and attitudinal change may enhance long-term program sustainability and youth engagement. Future research could also include objective measures such as accelerometry or fitness indicators at least in subsamples to triangulate findings and reduce potential common method variance. Future research may also benefit from longitudinal designs to examine how these variables interact over time, and from including qualitative data to better understand adolescents’ lived experiences related to physical activity and health behaviors. To reduce potential biases inherent in self-report measures and to improve methodological rigor, future studies might incorporate objective metrics such as accelerometry or fitness indicators at least within subsamples, as well as longitudinal designs to observe changes over time. Including qualitative approaches could also enrich understanding by capturing adolescents’ lived experiences related to physical activity and health behaviors. Additionally, adopting more inclusive designs that consider adolescents with disabilities or chronic conditions would align with the principle that physical literacy is a competence for all individuals and ensure that educational and research applications are genuinely accessible.

This study has several limitations. The cross-sectional design does not allow for causal interpretations of the observed relationships. Self-report-based data may have introduced subjective biases into the measurements. The purposive sampling of participants from a limited number of schools in Turkey restricts the generalizability of the findings and may overlook potential cultural or regional differences. Measurement invariance across subgroups (e.g., gender or age) was not tested, which prevented the examination of possible group differences. The short, single-time data collection period does not permit the investigation of longitudinal changes. Finally, adolescents with disabilities or chronic conditions were not included, which limits the inclusiveness of the findings.

Despite these limitations, the study has several notable strengths. First, the sample size was large (n = 750) and determined *a priori* via power analysis, enhancing statistical precision and the stability of parameter estimates. Second, participants were recruited from multiple schools across Türkiye, improving ecological validity and the relevance of the findings to real-world educational settings. Third, we used validated instruments with demonstrated reliability and construct validity in both prior adaptation studies and the current sample (e.g., satisfactory Cronbach’s alpha coefficients and acceptable CFA fit indices reported in the Methods section). Fourth, the hypothesized mediation model was tested using structural equation modeling with 5,000 bootstrap resamples, providing robust inferences for indirect effects. In addition, the balanced gender distribution and standardized, supervised in-class data collection procedures likely reduced procedural variability and supported data quality. Collectively, these strengths increase confidence in the internal consistency of the measures, the robustness of the mediation findings, and the practical utility of the results for school-based health promotion.

Nevertheless, it is equally important to acknowledge certain methodological constraints that may limit the generalizability and causal interpretation of the findings. However, given the cross-sectional design and purposive sampling, these conclusions should be interpreted as associations consistent with theoretical expectations rather than definitive causal evidence. Although the current study offers valuable insights, future research is needed to examine additional mediators and contextual factors such as peer dynamics, school climate, or digital media habits that may influence the relationship between physical literacy and health outcomes. Moreover, adopting inclusive designs that involve adolescents with disabilities or chronic conditions, and incorporating objective measures (e.g., accelerometry) alongside self-report data, could strengthen future investigations. Longitudinal and mixed-method designs could further enrich understanding by capturing change over time and the subjective experiences of adolescents. Overall, these findings carry important implications for educators, policymakers, and practitioners seeking to develop youth-centered strategies that not only teach health-related knowledge, but also cultivate positive attitudes and lifelong health habits.

## 6 Conclusion

This study demonstrated that physical literacy is positively associated with sustainable healthy lifestyle among adolescents, and that physical activity attitude plays a significant mediating role in this relationship. The findings indicate that physical literacy is a multidimensional competence that not only involves knowledge and skills but also shapes attitudes and behaviors. This underscores the importance of viewing physical literacy as a holistic construct that integrates cognitive, affective, and behavioral domains. Therefore, while developing physical literacy through educational and health policies, strategies aimed at transforming attitudes should also be planned simultaneously. Programs designed to promote healthy behaviors in youth should include components that foster motivation, confidence, and emotional engagement in addition to physical competence. The results contribute to the expansion of the theoretical framework in the existing literature and highlight the need for comprehensive intervention strategies to promote healthy lifestyles among young individuals. From a practical perspective, these findings suggest that schools, families, and community organizations should collaborate to design programs that integrate physical literacy with everyday lifestyle practices, such as active transportation, recreational sports, and digital health tools. Educators can adapt curricula to emphasize not only motor skills but also motivational and socio-emotional aspects, while policymakers may prioritize investments in supportive environments (e.g., safe playgrounds, inclusive facilities) that encourage sustainable healthy behaviors. Practitioners working in adolescent health promotion can use these insights to tailor interventions that address both cognitive understanding and emotional engagement, thereby increasing adherence and long-term impact. In particular, the study provides evidence to support integrative approaches in school-based health promotion, where physical education is aligned with behavioral science principles. These implications highlight that fostering positive physical activity attitudes may serve as a scalable strategy to bridge gaps between health knowledge and consistent health behavior adoption.

## Data Availability

The data presented in this study are available on request from the corresponding author. The data are not publicly available due to ethical and privacy restrictions.
